# Analysis of Static Plantar Pressures in School-Age Children with and without Functional Hallux Limitus: A Case-Control Study

**DOI:** 10.3390/bioengineering10060628

**Published:** 2023-05-23

**Authors:** Claudia Cuevas-Martínez, Ricardo Becerro-de-Bengoa-Vallejo, Marta Elena Losa-Iglesias, Israel Casado-Hernández, Oriol Turné-Cárceles, Laura Pérez-Palma, João Martiniano, Juan Gómez-Salgado, Daniel López-López

**Affiliations:** 1 Research, Health, and Podiatry Group, Department of Health Sciences, Faculty of Nursing and Podiatry, Industrial Campus of Ferrol, Universidade da Coruña, 15403 Ferrol, Spain; 2 Departament de Podologia, Facultat de Medicina i Ciències de la Salut, Universitat de Barcelona, 08036 Barcelona, Spain; 3 Facultad de Enfermería, Fisioterapia y Podología, Universidad Complutense de Madrid, 28040 Madrid, Spain; 4 Faculty of Health Sciences, Universidad Rey Juan Carlos, 28933 Alcorcón, Spain; 5 Escola Superior de Saúde da Cruz Vermelha Portuguesa, 1300-125 Lisbon, Portugal; 6 Department of Sociology, Social Work and Public Health, Faculty of Labour Sciences, University of Huelva, 21071 Huelva, Spain; 7 Safety and Health Postgraduate Programme, Universidad Espíritu Santo, Guayaquil 092301, Ecuador

**Keywords:** static, plantar pressure, foot pressure, children, hallux limitus

## Abstract

Background: The presence of hallux limitus in adulthood is frequently encountered in clinical practice, generating other biomechanical, structural, and functional compensations in dynamics secondary to blockage of the main pivot in the sagittal plane, the first metatarsophalangeal joint. In addition, the presence of functional hallux limitus (FHL) in school-age children is also increasing. Currently, there is a lack of scientific literature about this condition in the pediatric population, and early diagnosis is necessary to reduce future biomechanical disorders and avoid the development of foot arthritis. The purpose of this research was to identify static plantar pressures in school-age children with and without hallux limitus. Methods: A total sample of 106 children aged between six and twelve years old was divided into two groups: the case group (53 subjects with functional hallux limitus) and the control group (53 subjects without functional hallux limitus). Data were acquired with the participants in a standing barefoot position on the pressure platform, and the hallux limitus functional test was performed in a sitting position to classify the individuals into the determined study group. The variables analyzed in the research were: plantar pressure, bilateral forefoot and rearfoot surface area, bilateral forefoot and rearfoot ground reaction forces, bilateral forefoot and rearfoot distribution of body weight, total left and right surface area, maximum pressure of the left foot and right foot, medium pressure of the left foot and right foot, ground reaction forces of the left foot and right foot, and the weight of each foot. Results: Age was the only descriptive quantitative variable that showed a significant difference between the two study groups, with a *p*-value of 0.031. No statistically significant differences were found between groups in the bilateral forefoot and rearfoot surface area, ground reaction forces, distribution of body weight, or maximum and medium plantar pressure in the left and right foot. Conclusions: Changes in the location of the maximum pressure were observed, particularly in older participants with FHL, but these results were not significant. The findings of this study did not show significant differences between the static plantar pressures of school-age individuals with and without functional hallux limitus.

## 1. Introduction

Dorsiflexion (DF) limitation in the movement of the first metatarsophalangeal joint (MTPJ) is defined as hallux limitus (HL) [1,2]. HL is a common foot disorder suffered by the adult population, and it is characterized by an increase in incidence with age [3]. In addition, HL is the second most common cause of pathology related to the first ray and is characterized by a build-up of peak plantar pressure in the hallux instead of the first metatarsal head, which is greater and occurs at a faster rate [4,5]. One of the main biomechanical characteristics of HL is the first MTPJ blockage in the sagittal plane [6]. This foot disorder is one of the main causes of the premature development of foot arthritis, which occurs in 1 in 40 subjects over the age of 50 [3,7,8]. The first MTPJ plays a fundamental role in gait patterns [9], specifically in the terminal stance and pre-swing gait phases. When the center of gravity is changing to propel the body forward, the first MTPJ has to provide suitable dorsiflexion, which is necessary to generate the correct moment of propulsion [2,10,11]. The correct first MTPJ range of motion (ROM) is between 50° and 90°, and as a result, the joint has to support from 40% to 60% of the body weight [3,12,13,14]. 

Previous studies correlated a limitation of the first MTPJ dorsiflexion movement with pronated or very pronated foot development because of biomechanical maladjustment and structural blockage that decrease movement [1,15,16,17] accompanied by an incorrect windlass mechanism [2].

According to previous research, there is a significant correlation between subjects who have developed HL and plantar fascia tightness. Subjects whose extension of the hallux is decreased usually present higher tension in the plantar fascia compared to subjects without HL [2].

This limitation prior to the development of HL is called functional hallux limitus (FHL) [18], which can be diagnosed clinically in the pediatric population [11,19,20]. The movement of FHL is restricted in a closed kinetic chain [21] and is characterized by limitations in the movement of the first MTPJ in the dynamic gait stage [17].

Portable pressure platforms are devices used to evaluate plantar pressure in stance and dynamic gait patterns with high reliability in the data collected, which is more reliable in subjects in stance situations than in different gait phases due to variability in dynamic gait [22].

However, to the best of our knowledge, there are no previous studies to determine the surface area, average peak pressure, and body weight of the lower limbs regarding this foot pathology in the pediatric population.

The purpose of this research was to analyze the static plantar pressure variation in school-age individuals with and without functional hallux limitus.

Our hypothesis was that school-age subjects with hallux limitus would have increased plantar pressures.

## 2. Material and Methods

### 2.1. Study Design

A case-control study was developed between January 2022 and February 2023. The recruitment of the participants was carried out in multiple centers in different places in Spain and was performed consecutively and conveniently using the same protocol and platform.

This study followed the Strengthening the Reporting of Observational Studies in Epidemiology (STROBE) guidelines [23]. This case-control study assessed the static plantar pressures in school-age children with and without functional hallux limitus.

This research was approved by the Bioethics Committee of the University of Barcelona on 21 December 2021, with ID: IRB00003099. It complied with all current regulations on human experimentation, as well as the Declaration of Helsinki and Organic Law 3/2018, of 5 December, on the protection of personal data and the guarantee of digital rights [24].

All the subjects were previously screened by an expert podiatrist with 10 years’ experience. For this study, the following inclusion criteria were met: (1) to be healthy subjects without musculoskeletal or neurological diseases; (2) to be without foot disorders and with functional hallux limitus; (3) to be more than 4 years old and less than 12 years old; and (4) to have given their assent and whose parents or guardians had signed the informed consent form. The exclusion criteria were: (1) to have hypermobility syndrome; (2) to have a neurological disease; (3) to have previous orthopedic history affecting one or both lower extremities; (4) to have rheumatic pathologies; (5) to have a first MTPJ angular value of less than 10° with ankle flexion and less than 10° with the knee in extension; (6) to be older than 12; and (7) to refuse to participate and have the consent form signed by their parents or guardians. FHL was evaluated by an expert podiatrist with more than 10 years’ experience. For diagnostic purposes, FHL was defined as restricted movement in the first MYPJ in a closed kinetic chain [21], and this pathology was diagnosed by performing the FHL test [18].

To calculate the sample size, the specific levels of confidence, power, and groups of equal size were applied using Epidat version 4.2 software (Consellería de Sanidade, Xunta de Galicia, Spain; Pan American Health Organization (PAHO-WHO); University CES, Colombia). To accomplish this with statistical confidence, an 80% statistical power analysis with a β error of 20%, an α error of 0.05, and a two-tailed test were required. A total sample size of 106 children aged between six and twelve years was included in the study. The subjects were divided into two groups: 53 with FHL and 53 healthy subjects.

### 2.2. Method

The functional hallux limitus test was performed with the patient in a sitting or supine position, and the investigator applied force below the first metatarsal head (MTH) with the passive and active hands, performing a DF of the proximal phalanx of the first toe. When there was no limitation, the force applied under the first MTH was approximately the same as used to perform the DF of the proximal phalanx, which allows a DF of the first MTPJ, plantar flexion (PF) of the first MTH, pronation of the forefoot (FF), supination of the rearfoot (RF), and activation of the windlass mechanism, a series of movements necessary for effective and non-injurious propulsion, which was considered a negative test (HLF -). In a foot with HLF, the applied forces were not balanced, being greater than that necessary to perform the DF of the first MTPJ than that applied under the first MTH [18], and represented a positive HLF test (HLF +).

Data acquisition was performed using a laptop linked via USB to a portable pressure platform. The commercially available software program T-Plate^®^ for Windows was used to collect and manage the data. Autocalibration was performed before each use. The static plantar pressure was measured by the same clinician.

**Figure 1:**
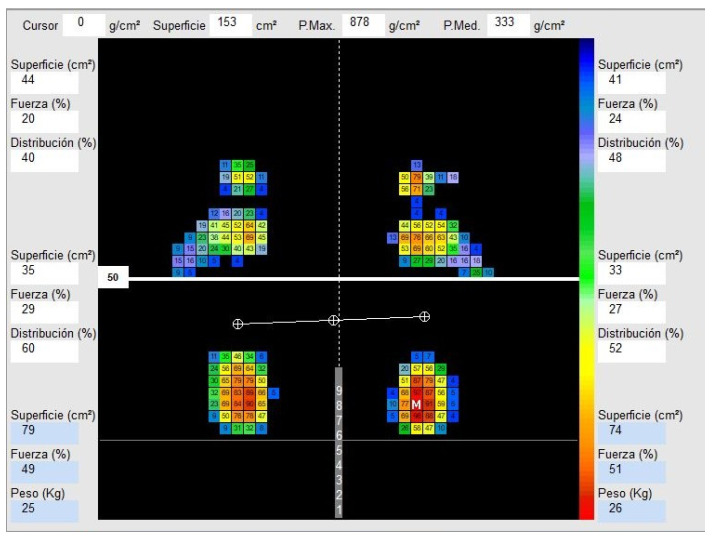
This figure shows the plantar pressure data regarding surface, force, and distribution from the forefoot and rearfoot areas and the percentages between areas and feet.

The outcome measurements for subjects diagnosed with functional hallux limitus and healthy matched-paired controls included static plantar measurements: plantar pressure, bilateral forefoot and rearfoot contact surfaces, bilateral forefoot and rearfoot ground reaction forces, bilateral forefoot and rearfoot distribution of body weight, total left and right surface area, maximum pressure of the left foot and right foot, medium pressure of the left foot and right foot, ground reaction forces of the left foot and right foot, and the weight of each foot (Figure 1).

During the static plantar pressure measurement assessment, the participant was standing barefoot on the plantar pressure platform in a relaxed position with the arms at the sides of the trunk. The plantar foot measurements were simultaneously collected for both feet. If the participant moved during testing, the data were discarded and the trial was repeated.

Three static analyses of each participant were performed in order to have a more reliable and representative result. The total time for each data acquisition of the subject in a static position was 30 s. If any analysis was very dispersed, it was classified as an outlier, and a new analysis was performed on the participant.

**Figure 2:**
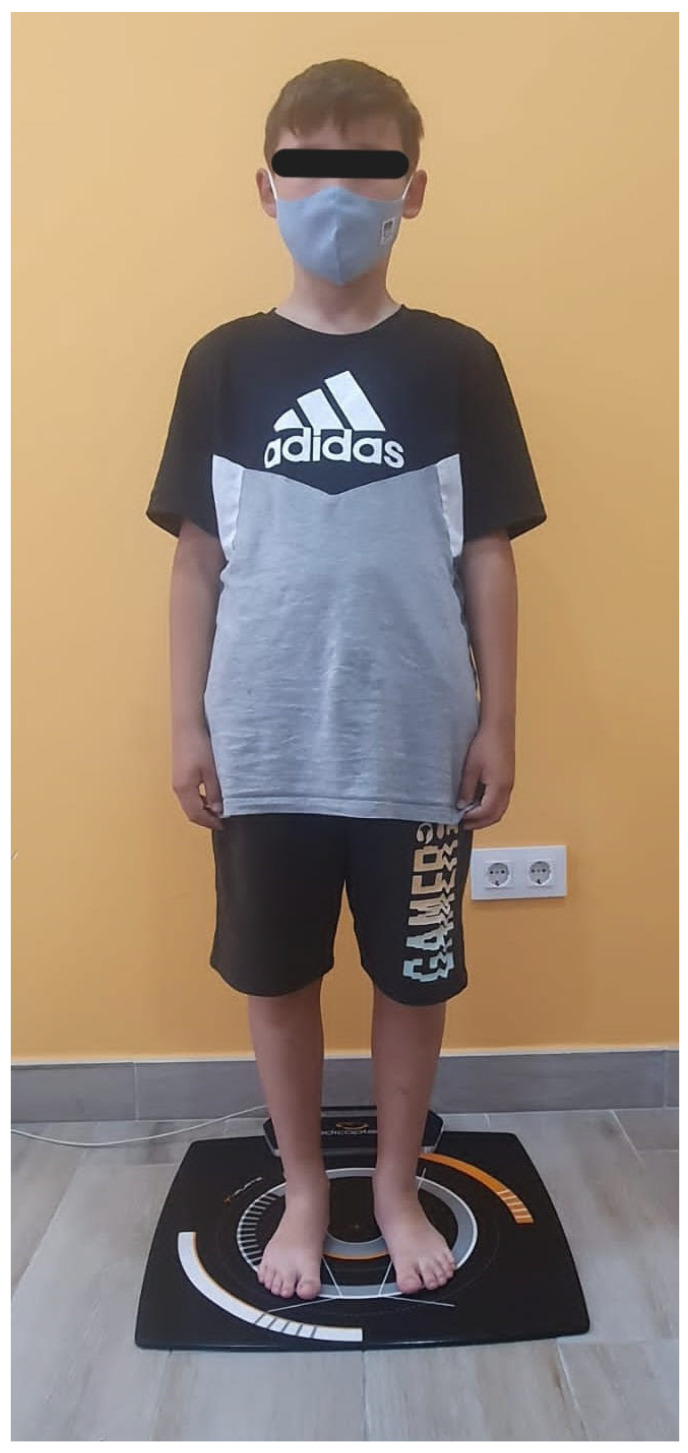
Subject in a relaxed standing position during the acquisition of plantar pressure data from the portable pressure platform.

When the participant was uncomfortable or restless, the measurements were discarded (Figure 2).

The output software included mapping pressures, which provided the plantar pressure magnitude of the forefoot and rearfoot surface area of each foot, forefoot and rearfoot ground reaction forces (GRF) of each foot (percentage), forefoot and rearfoot distribution of body weight of each foot (percentage), surface area of each foot (square centimeters), maximum plantar pressure of each foot (grams/square centimeters), medium plantar pressure of each foot, GRF of each foot (percentage), and weight of each foot (kilograms).

### 2.3. Statistical Analysis

The statistical analyses were performed using the Statistical Package for the Social Sciences (SPSS software, version 19.0). Parametric data were described as mean ± standard deviation (SD) and range (minimum–maximum values). Normality was checked with the Kolmogorov-Smirnov test on the variables studied (*p* > 0.05) in the data on static plantar measurements. Independent t-tests were used for the outcome variables that were normally distributed. The non-parametric Mann-Whitney “U” test was performed to consider contrasts among the two groups with or without HLF.

In all analyses, *p* < 0.05 (with a 95% confidence interval) was considered statistically significant.

## 3. Results

### 3.1. Sociodemographic and Descriptive Data

Out of the 106 recruited participants, only 53 were diagnosed with FHL, and the other 53 were healthy controls. The sample size was composed of 55 boys and 51 girls. The ages of the participants were between 6 and 12 years old. No significant differences were observed between groups in relation to the quantitative sociodemographic and descriptive data, just in the age data (Table 1).

Age was the only descriptive quantitative variable that showed a significant difference between the two study groups, with a *p*-value of 0.031. The remaining values were not significant enough to determine differences between the study groups. This indicates that there was no relationship between the presence or absence of an FHL and weight, height, BMI, sex, or foot size. Foot number size was the next most significant value, although it was not enough to establish a causal relationship with FHL, showing a *p*-value of 0.099.

### 3.2. Primary Outcome Measures

The primary outcome measurements for subjects diagnosed with functional hallux limitus, healthy matched-paired controls, and the total sample are presented in Table 2. The static plantar pressure results showed no significant differences between groups.

No statistically significant differences were found between groups in the bilateral forefoot and rearfoot surface area, bilateral forefoot and rearfoot GRF, bilateral forefoot and rearfoot distribution of body weight, total left and right surface area, maximum pressure of the left foot and right foot, medium pressure of the left foot and right foot, ground reaction forces of the left foot and right foot, and the weight of each foot.

## 4. Discussion

This is the first study to relate FHL disorders to plantar pressure in a sample of school-age subjects. The aim of this study was to analyze the plantar pressure in a stationary position in subjects between 4 and 12 years old, with and without functional hallux limitus.

Functional hallux limitus disorder is characterized by a limitation of movement in the first MTPJ in the dynamic gait stage. A previous study performed by Gatt et al. corroborated the relationship between subjects with pronated feet and the dorsiflexion movement limitation in the gait and concluded that the greater the pronation of the foot in the gait, the less the first MTPJ dorsiflexion [16]. In our research, subjects were in a static position with normal pronation of the foot, and no changes in the plantar pressures were shown, besides no changes in the surface areas and forces. We found discretely higher data in the left foot between groups, but it was not statistically significant.

The research conducted by Halstead et al. analyzing the clinical test of dorsiflexion of the hallux in a passive way in stance position concluded that there were no variations in the maximum dorsiflexion performed in a relaxed weight-bearing static position and walking [25]. Our research was performed with the subjects in a static position, and the dorsiflexion MTFJ test was also realized in a static position.

According to the research by Aquino et al., excessive pronation did not always affect the establishment of the windlass mechanism in adulthood because the musculoskeletal function may be correct but not the joint movement of the first MTPJ [26].

In addition, the research performed by Durrant on school-age subjects concluded that the GFR, which acts on the foot, was relatively small owing to the low weight of some subjects and could not be captured by a pressure platform. In addition, dynamics occur when different compensatory mechanisms generated by this functional limitation are activated. Non-significant quantitative results were obtained between the two study groups, and FHL occurred in dynamics when different compensatory mechanisms generated by this functional limitation were activated [20]. In our research, the portable plantar pressure device proved to be a reliable system for plantar pressure acquisition [22].

Although previous studies have reported a limitation of the DF movement of the first MTPJ associated with a pronated or very pronated foot due to biomechanical alterations [1,15,16,17], currently there is very little consensus on the pathophysiology, cause, and treatment of HL in adults and younger children [3,5,27,28,29,30]. The authors do agree that the etiology of HL is multifactorial [8,31,32,33,34].

According to our research, the quantitative variables included in Table 2 did not show a significant difference between the two study groups. This may be associated with alterations in joint mobility of the foot and postural alterations in subjects with FHL.

Although there are several studies and reviews related to foot posture and plantar pressure in adults [35,36,37], currently there is little research on the relationship between pathologies that affect the school-age population and the consequent biomechanical alterations, anomalies in static plantar pressures [38], and subsequent pathologies to help avoid harmful biomechanical compensation in the long term.

Regarding our initial hypothesis, no statistically significant differences were shown between static plantar pressures in school-age children with and without FHL. However, in the images obtained on the pressure platform, in individuals of greater age and, therefore, greater weight and GRF, a smaller contact area was observed in the forefoot and greater pressure under the first MTH in the feet with HLF+. In subjects without HLF, the forefoot support surface was broader and more diffuse (Figure 1). No significant results were obtained in this data collection, perhaps because the ages and weights of the subjects were dispersed owing to the wide range of characteristics of the individuals studied.

Therefore, other reference parameters may be necessary to compare these values.

The ROM of the first MTPJ in static conditions is higher than in dynamic conditions, so static measurements may not be a good assessment to determine the functionality of the foot in dynamic pathologies [20].

This study has several limitations. First, the consecutive sampling method could be replaced by random sampling in the future. Second, the age range is wide, and for future research, the increase in the sample of each age group could be assessed to evaluate the range in which significant results begin to be obtained between both study groups, as well as the influence of overweight and obesity on the plantar pressure values.

In addition, the study of dynamic plantar pressures in school-age children with and without FHL is necessary since the GFRs are higher in dynamic situations and significantly different results may be obtained between both groups.

In future research, it would be interesting to study dynamic plantar pressures in school-age children with and without functional hallux limitus.

## 5. Conclusions

Although changes in pressure were observed, the results of the research did not show significant differences between the plantar pressures of school-age individuals with and without FHL. However, in the images viewed and obtained individually, changes in the location of the maximum pressure were observed, particularly in older participants with FHL, which was located below the first toe. These results were not significant enough to obtain valid results in this study.

## Figures and Tables

**Table 1:** Quantitative sociodemographic and descriptive data for subjects diagnosed with functional hallux limitus, healthy controls, and the total sample.

Quantitative Descriptive Data	Total Group (*n* = 106) Mean ± SD (Range)	FHL (*n* = 53) Mean ± SD (Range)	Healthy (*n* = 53) Mean ± SD (Range)	*p*-Value
Age (years)	9.47 ± 1.501 (6–12)	9.81 ± 1.27 (8–12)	9.13 ± 1.64 (6–12)	0.031 ^†^
Weight (kg)	36.38 ± 11.31 (17.95–90.00)	36.69 ± 8.50 (22.00–56.60)	36.98 ± 13.63 (17.95–90.00)	0.542 ^†^
Height (cm)	140.14 ± 11.44 (113.00–176.00)	141.26 ± 9.50 (121.00–167.00)	139.02 ± 13.10 (113.00–176.00)	0.231 ^†^
BMI (kg/m^2^)	18.50 ± 3.91 (11.00–40.00)	18.11 ± 2.81 (14.00–25.00)	18.89 ± 4.77 (11.00–40.00)	0.729 ^†^
Sex (male/female)	55/51 (51.9/48.1)	23/30 (43.4/56.6)	32/21 (60.4/39.6)	0.120 ^‡^
Foot Size	35.63 ± 3.58 (15.00–44.0)	36.18 ± 2.38 (30.0–43.0)	35.09 ± 4.42 (15.00–44.0)	0.099 ^†^

**Abbreviations:** kg, kilogram; cm, centimeter; m^2^, square meter; %, percentage; SD, standard deviation; *n*, number. ^†^ Mann-Whitney U test was used. ^‡^ Fisher’s exact test was used. In all the analyses, *p* < 0.05 (with a 95% confidence interval) was considered statistically significant.

**Table 2:** Outcome measurements for subjects diagnosed with functional hallux limitus, healthy matched-paired controls, and the total sample.

Quantitative Descriptive Data	Total Group (*n* = 106) Mean ± SD (Range)	FHL (*n* = 53) Mean ± SD (Range)	Healthy (*n* = 53) Mean ± SD (Range)	*p*-Value
Left forefoot surface area (cm^2^)	31.36 ± 10.83 (6–66)	31.85 ± 9.39 (14–64)	30.87 ± 12.17 (6–66)	0.450 ^†^
Right forefoot surface area (cm^2^)	30.08 ± 12.33 (0–76)	30.06 ± 11.54 (8–76)	30.11 ± 13.18 (0–66)	0.889 ^†^
Left rearfoot surface area (cm^2^)	32.46 ± 10.04 (17–70)	32.66 ± 9.49 (17–55)	32.26 ± 10.65 (19–70)	0.565 ^†^
Right rearfoot surface area (cm^2^)	29.95 ± 10.51 (12–64)	29.11 ± 8.77 (15–49)	30.79 ± 12.03 (12–64)	0.697 ^†^
Left forefoot force (%)	20.17 ± 7.12 (3–46)	20.58 ± 6.50 (7–39)	19.75 ± 7.72 (3–46)	0.360 ^†^
Right forefoot force (%)	18.33 ± 6.80 (0–38)	17.81 ± 6.20 (5–38)	18.85 ± 7.37 (0–33)	0.233 ^†^
Left rearfoot force (%)	33.50 ± 8.49 (17–63)	33.42 ± 7.49 (17–51)	33.58 ± 9.45 (17–63)	0.907 ^†^
Right rearfoot force (%)	27.98 ± 7.07 (6–48)	28.27 ± 6.46 (15.00–42)	27.70 ± 7.69 (6–48)	0.716 ^†^
Left forefoot distribution (%)	37.79 ± 12.41 (5–72)	38.19 ± 11.25 (15–66)	37.40 ± 13.57 (5–72)	0.704 ^†^
Right forefoot distribution (%)	39.40 ± 13.77 (0–79)	38.47 ± 11.79 (12–71)	40.32 ± 15.56 (0–79)	0.349 ^†^
Left rearfoot distribution (%)	62.21 ± 12.41 (28–95)	61.81 ± 11.25 (34–85)	62.60 ± 13.57 (28–95)	0.704 ^†^
Right rearfoot distribution (%)	59.75 ± 14.80 (7–100)	59.66 ± 14.21 (7–88)	59.85 ± 15.50 (21–100)	0.752 ^†^
Left surface area (cm^2^)	64.10 ± 18.87 (26–136)	64.89 ± 15.92 (31–91)	63.32 ± 21.55 (26–136)	0.321 ^†^
Right surface area (cm^2^)	60.13 ± 20.33 (14– 130)	59.17 ± 17.11 (32–107)	61.09 ± 23.24 (14–130)	0.899 ^†^
Left maximum plantar pressure (kPa)	813.63 ± 212.67 (73–1357)	816.19 ± 184.59 (360.10–1357)	811.06 ± 239.25 (73–1217)	0.830 ^†^
Right maximum plantar pressure (kPa)	754.59 ± 185.56 (332.42–1424.00)	783.28 ± 183.47 (484.40–1357.00)	725.91 ± 184.89 (332.42–1424.00)	0.129 ^†^
Left medium plantar pressure (kPa)	314.88 ± 64.80 (146.00–506.00)	314.50 ± 60.15 (182.50–506.00)	315.26 ± 69.72 (146–463.00)	0.691 ^†^
Right medium plantar pressure (kPa)	323.21 ± 333.54 (151.00–3671.00)	294.21 ± 57.25 (191–437)	352.21 ± 468.67 (151.00–3671.00)	0.793 ^†^
Left force (%)	53.81 ± 6.72 (34.00–70.00)	54.08 ± 6.37 (42–70)	53.55 ± 7.10 (34.00–69.00)	0.628 ^†^
Right force (%)	46.28 ± 6.72 (30–66)	45.92 ± 6.37 (30–58)	46.64 ± 7.09 (31–66)	0.498 ^†^
Left weight (kg)	19.76± 6.25 (9–44)	19.96 ± 5.60 (9–31)	19.57 ± 6.89 (11–44)	0.350 ^†^
Right weight (kg)	17.11 ± 6.29 (6–46)	16.83 ± 4.63 (9–26)	17.40 ± 7.62 (6–46)	0.730 ^†^

**Abbreviations:** kPa, kilopascals; kg, kilogram; cm, centimeter; cm^2^, square centimeter; %, percentage; SD, standard deviation; *n*, number. ^†^ Mann-Whitney U test was used. In all the analyses, *p* < 0.05 (with a 95% confidence interval) was considered statistically significant.

## Data Availability

The dataset supporting the conclusions of this article is available upon request at claudia.cuevas@udc.es, Research, Health, and Podiatry Group (Department of Health Sciences, Faculty of Nursing and Podiatry, Industrial Campus of Ferrol, Universidade da Coruña).
